# Effects of Physical Exercise and Motor Activity on Oxidative Stress and Inflammation in Post-Mastectomy Pain Syndrome

**DOI:** 10.3390/antiox12030643

**Published:** 2023-03-04

**Authors:** Marco Calapai, Luisa Puzzo, Giuseppe Bova, Daniele Alfio Vecchio, Rosario Blandino, Alessia Barbagallo, Ilaria Ammendolia, Luigi Cardia, Maria De Pasquale, Fabrizio Calapai, Emanuela Esposito, Fabio Trimarchi, Debora Di Mauro, Gioacchino Calapai, Carmen Mannucci

**Affiliations:** 1 Breast Unit, San Vincenzo Hospital, Azienda Sanitaria Provinciale Messina, 98039 Messina, Italy; 2 Pain Therapy Unit, San Vincenzo Hospital, Azienda Sanitaria Provinciale Messina, 98039 Messina, Italy; 3 Department of Clinical and Experimental Medicine, University of Messina, 98125 Messina, Italy; 4 Department of Chemical, Biological, Pharmacological and Environmental Sciences, University of Messina, 98125 Messina, Italy; 5 Department of Human Pathology of Adult and Childhood “Gaetano Barresi”, University of Messina, 98125 Messina, Italy; 6 Genetics and Pharmacogenetics Unit, A.O.U. “G. Martino”, University of Messina, 98125 Messina, Italy; 7 Department of Biomedical and Dental Sciences and Morphological and Functional Imaging, University of Messina, 98125 Messina, Italy

**Keywords:** Post-Mastectomy Pain Syndrome, pain, physical exercise, inflammation, oxidative stress

## Abstract

It is estimated that 10–50% of interventions can generate persistent post-surgical pain. Chronic post-mastectomy pain is a condition persisting for at least three months after surgery. It has been shown that physical activity in the cancer patient allows the improvement of the pain symptom. The aim of this study was to evaluate the effects of physical activity on the intensity and interference of chronic pain in the quality of life of women underwent mastectomy needed for breast cancer removal. The secondary objective was to measure the effects of physical activity on inflammatory and oxidative markers in the same population. A Numeric Rating Scale (NRS) was used to assess pain intensity, and Brief Inventory Pain (BIP) was used for assessing interference of pain in quality of life. Physical activity was measured with the International Physical Activity Questionnaire (IPAQ). Inflammatory mediators such as interleukin (IL)-6, IL-8, tumor necrosis factor (TNF)-α, c-reactive protein (CRP), and biomarkers of oxidative stress malondialdehyde (MDA), superoxide dismutase (SOD), and catalase (CAT) were evaluated in the blood of patients. All the evaluations were performed after three and six months after surgery. Results showed that adequate physical activity can diminish intensity and interference of pain and that these effects are associated with a reduction of blood biomarkers of inflammation.

## 1. Introduction

Pain is an unpleasant sensation common to people who undergo surgery. Several studies indicate that 40–60% of patients experience post-operative pain and that it can be caused by both internal and external stimuli [1]. Most patients define the post-operative experience as a very painful condition that interferes with normal daily activities [2]. Chronic pain is a common, complex, and distressing problem that has a profound impact on individuals and society [3]. Chronic pain is a pain that persists past normal healing time and generally lasts or recurs for more than 3 to 6 months. It can become chronic in a broad spectrum of conditions, including surgical interventions. In fact, it is estimated that between 10 and 50% of elective interventions can generate persistent post-surgical pain [4,5].

Chronic pain can be present after surgical intervention needed to remove breast cancer. In many people with chronic non-specific pain, central nervous system sensitization can explain sufference from pain in the absence of a clear origin of nociceptive input or in the absence of enough tissue damage to explain the experienced pain severity, disability and other symptoms. Central sensitizitation is defined as an amplification of neural signalling within the central nervous system that elicits pain hypersensitivity [6]. The need for early recognition of central sensitization in patients with chronic pain was raised by the International Association for the Study of Pain (IASP), who recently introduced the term “nociplastic pain” as a third mechanistic pain descriptor in addition to nociceptive (pain attributable to the activation of the peripheral receptive terminals of primary afferent neurones in response to noxious chemical, mechanical, or thermal stimuli) and neuropathic (pain caused by a primary lesion or disease of the somatosensory nervous system) pain [7]. Nociplastic pain is defined by the IASP as “pain that arises from altered nociception despite no clear evidence of actual or threatened tissue damage causing the activation of peripheral nociceptors or evidence for disease or lesion of the somatosensory system causing the pain” [8].

Chronic post-mastectomy pain is a condition characterized by pain in the anterior chest, armpit, and/or upper arm, usually ipsilateral to surgery, which begins after mastectomy or quadrantectomy and persists for longer three months after surgery [9,10]. After breast surgery, women can develop severe post-surgery pain that becomes chronic post-mastectomy pain (PMP); 25% to 60% of women who have been subjected to mastectomy for breast cancer suffer from post-surgery moderate-to-severe pain [11]. Chronic PMP is the main symptom of a syndrome referring to the occurrence of pain in and around the area of mastectomy lasting beyond three months after surgery when all other possible causes of pain are ruled out. It may last for years or be present for life [12].

Post-mastectomy consequences can require early rehabilitation. The former appear in the first months and include nerve, muscular, joint and vascular lesions, shoulder impairment, axillary web syndrome, intercostal brachial nerve syndrome, winged scapular, phlebitis, thrombophlebitis, lymphangitis, and transitory edemas [13]. It has been observed that early assisted mobilization and home rehabilitation, associated with written information on precautionary hygienic measures to observe, can play a crucial role in reducing the occurrence of post-operative side effects of upper-limbs in patients subjected to mastectomy and reconstructive surgery [14].

The benefits of physical activity on the general population have been extensively studied. It has been shown that physical activity in the cancer patient allows for the recovery of the previous functional capacities, strength and flexibility, improvement of the pain symptom and asthenia, as well as the reduction of alterations in the hematological picture such as neutropenia, anemia, and thrombocytopenia [15].

The relation between oxidative stress status and nociception has been evident for decades. Following inflammatory stimuli, the formation of reactive oxygen species (ROS) increases and is involved in the regulation of immune response [16]. Moreover, it has been hypothesized that while inflammation and oxidative stress play a role in the acute nociceptive phase, ROS formation could be principally responsible for the perpetuation of chronic pain [17]. On the basis of these observations, the potential role of exercise and motor activity on chronic pain, inflammatory markers, and oxidative stress has been investigated in a prospective observational study recruiting women affected by PMP. The aim of this study was to evaluate the effects of physical activity on the intensity and interference of chronic pain in the quality of life of women underwent mastectomies. The secondary objective of this study was to measure the effects of physical activity on inflammatory and oxidative markers.

## 2. Materials and Methods

### 2.1. Study Design

A prospective observational unicentric cohort study was performed by recruiting female patients who had undergone a unilateral or bilateral mastectomy due to resection of stage II and III breast cancer not yet followed by breast reconstruction, chemotherapy, and radiation who were aged 18 years or over. One hundred and fifty-eight (158) female patients were screened for the study. Pain assessment and the motor activity of each participant in the study was measured at 3 and 6 months after the surgical intervention by verbal administration of the following questionnaires: Numeric Rating Scale (NRS), used to assess pain intensity, and Brief Inventory Pain (BIP) for assessing, together with pain intensity, the interference of pain in quality of life. Physical activity was measured with the International Physical Activity Questionnaire (IPAQ). At the same timepoints, blood biomarkers of pain such as interleukin (IL)-6, IL-8, tumor necrosis factor alpha (TNF-α), c-reactive protein (CRP), and blood biomarkers of oxidative stress malondialdehyde (MDA), superoxide dismutase (SOD), and catalase (CAT) were evaluated.

### 2.2. Participants

Participants were enrolled during the years 2021 and 2022 at the Breast Unit of San Vincenzo Hospital of Taormina in collaboration with the Azienda Ospedaliera Universitaria (AOU) Policlinico “G. Martino” of Messina, Italy. The following inclusion criteria were adopted: women aged greater than 18 years with a diagnosis of prior Phase II or III breast cancer who underwent mastectomy due to cancer removal three months ago.

The exclusion criteria were chemotherapy and radiation during the six months following surgery, medical history of other cancer types, immune system disorders (multiple sclerosis, HIV, lupus), rheumatic diseases, fibromyalgia, and recent symptoms of flu (cough, fever). Women with breast cancer at Stages 0 and I were excluded due to possible and frequent absence of pain. Female patients with cancer at Stage IV were excluded, as pain can be originated from any metastases. Women reporting pain before surgery and those co-affected by other kinds of tumors or by other pathologies characterized by chronic pain were excluded. According to the above-described inclusion and exclusion criteria, one hundred and twenty-six (126) patients were recruited for the study. All the patients were asked to sign the informed consent form to participate in the study. The study was approved by the Ethics Committee of AOU Policlinico “G. Martino”: Approval Number: Prot. 44-21, 22/03/2021, Board Name: Comitato Etico Interaziendale Messina. The trial was conducted according to the ethical principles of the Declaration of Helsinki and Good Clinical Practice principles were adopted. The sample size to enroll subjects in the study was calculated by Clincalc software. (ClinicalTrials.gov Identifier: NCT05713786).

### 2.3. Methodology

#### 2.3.1. Demographic and Surgical Variables 

Demographic variables that have been found to impact pain-related experiences, either in breast cancer or in the broader pain literature, including age, marital status, and level of education, were considered. The Italian education system includes primary (five years), secondary (three years), post-secondary (five years), and graduation stages (three-six years). Information about the lymph nodes dissection was collected for each participant. All the participants were Caucasian.

#### 2.3.2. Numerical Rating Scale (NRS)

The numeric rating scale (NRS) is a pain screening tool and a standard instrument in chronic pain studies commonly used to assess pain severity [13,18]. For the NRS, it has become important to define the level of change that better represents a clinical improvement [14,19]. NRS is a 0–11 point-scale where the end points are the extremes of no pain (point 0) and the worst pain is as bad as it could be (point 10) [15,20]. Through medical history (pain lasting three months after surgery) and their NRS score, the sample of women was divided in two groups: the PMP group, composed of women affected by pain and totalizing ≥ 5, and the Non-PMP group, composed of women totalizing <5. NRS was self-administered after six months.

#### 2.3.3. Brief Pain Inventory (BPI)

The BPI—Short Form is a 9-item self-administered questionnaire used to evaluate the severity of a patient’s pain and the impact of this pain on the patient’s daily activities [16,17,21,22]. Each item is scored on a 10-point numeric rating scale where 0 = no interference/pain and 10 = severe or complete interference/pain. A combination of items provides a pain severity subscale, assessing pain as “worst”, “least”, “average”, and “current pain”, and interference subscale, reporting the interference of pain with general activities [18,19,23,24]. BPI was self-administered three and six months after breast surgery.

#### 2.3.4. International Physical Activity Questionnaire (IPAQ)

Self-reported physical activity data were collected using the International Physical Activity Questionnaire (IPAQ) administered three and six months after surgery. The IPAQ questionnaire measures the type and amount of physical activity that is normally done. It addresses the number of days and time spent on physical activity (PA) in moderate intensity, vigorous intensity, and walking of at least 10-min duration over the last 7 days, and includes time spent sitting on weekdays over the last 7 days. The IPAQ comprises four different detailed PA levels (work-related activity, leisure-time activity, transport-related activity, and domestic activities), each with three intensities: walking, moderate, and vigorous. The total weekly physical activity was estimated by weighting time spent in each activity intensity with its estimated metabolic equivalent energy expenditure (Metabolic equivalent of task; MET) [20,25]. According to the questionnaire, women are classified into three categories: inactive, if they presented a value of METs less than 700, adequately active with METs between 700 and 2519, and highly active with METs > 3000.

#### 2.3.5. Biomarkers of Oxidative Stress and Inflammation

Serum levels of biomarkers of oxidative stress and inflammation were evaluated 3 and 6 months after surgery according to the manufactured protocol of commercial kits. Specifically, the following kits were used: SOD, Cayman No. 77547; CAT, Invitrogen (Thermofisher Scientific) #9876; MDA, Biocompare No. 5643; IL-6, IL-8, TNF-α, Diaclone s.a.s, n. 950.030.096, 950.050.096, and 950.090.096, respectively. CRP blood levels were quantified by flow cytometry.

#### 2.3.6. Statistics 

The Mann–Whitney U test or Wilcoxon test were used to evaluate the difference among the groups. Data are presented as mean ± standard error and *p* value < 0.05 was considered statistically significant. Spearman’s rank correlation was for correlation between biomarkers of inflammation and oxidative stress and NRS or BPI or IPAQ. According to the test, the correlation is considered “very weak for values between 0.00–0.19; “weak” for values between 0.20–0.039; “moderate” for values between 0.40–0.59; “strong” 0.60–0.079; and “very strong” for values between 0.80–1.0.

## 3. Results

One hundred and twenty-six (126) women who underwent a mastectomy during the years 2021 and 2022 were enrolled for the study. The mean age was 55.2 ± 9.8 years (range 32–71 years; median age 56 years). The mean BMI was 22.31 ± 1.5.

### 3.1. Numerical Rating Scale Score (NRSs) and Brief Pain Inventory (BPI)

After clinic examination, and according to the evaluation of NRS results collected 3 months after surgery, it was found that 47% (n = 58) of the patients enrolled for the study were affected by PMP Syndrome (PMP group), while 54% (n = 68) did not report any significant pain (Non-PMP group) (Table 1). On this basis, participants were divided into two groups, one called PMP and the other called non-PMP. The use of drugs that potentially influenced pain perception, such as tamoxifen and aromatase inhibitors, anti-inflammatory drugs, and corticosteroids, were equally distributed in the two different groups. In Table 1, age and percentage of surgical variables of women of the two groups are reported. In the group of PMP patients, the BPI score, evaluated 3 months after surgery, showed a statistically significant increase of pain intensity and interference in daily activities in PMP women compared with the non-PMP group (Table 2). Similar results were detected in both PMP and non-PMP groups. PMP pain was not associated with any demographic or surgery-related variables taken into account (Table 1).

### 3.2. Biomarkers of Oxidative Stress and Inflammation

All the biomarkers of inflammation (CRP, IL-6, IL-8, and TNF-α) were significantly increased in PMP women compared to non-PMP women either 3 or 6 months after surgery (Table 2). Biomarkers of oxidative stress showed a statistically significant decrease of SOD and CAT activity and a statistically significant increase of MDA levels in PMP women compared to non-PMP women (Table 2).

### 3.3. IPAQ Score

The evaluation of physical activity at IPAQ among the 58 women affected by PMP showed that 26 of them were categorized as inactive (<700 METs) and 38 were categorized as adequately active (>700 METs). No active women (>2510 METs) were present in the group. Moreover, NRS and BPI evaluated in PMP women showed a statistically significant increase (*p* < 0.01) of intensity and interference of pain in inactive women compared to adequate active women both 3 and 6 months after surgery (Table 3). Moreover, all the biomarkers of inflammation (CRP, IL-6, IL-8, and TNF-α) were significantly reduced in PMP women categorized as adequately active compared to inactive women either 3 or 6 months after surgery, while biomarkers of oxidative stress were not affected by physical activity 3 or 6 months after mastectomy (Table 4).

### 3.4. Spearman’s Correlation

**Figure 1:**
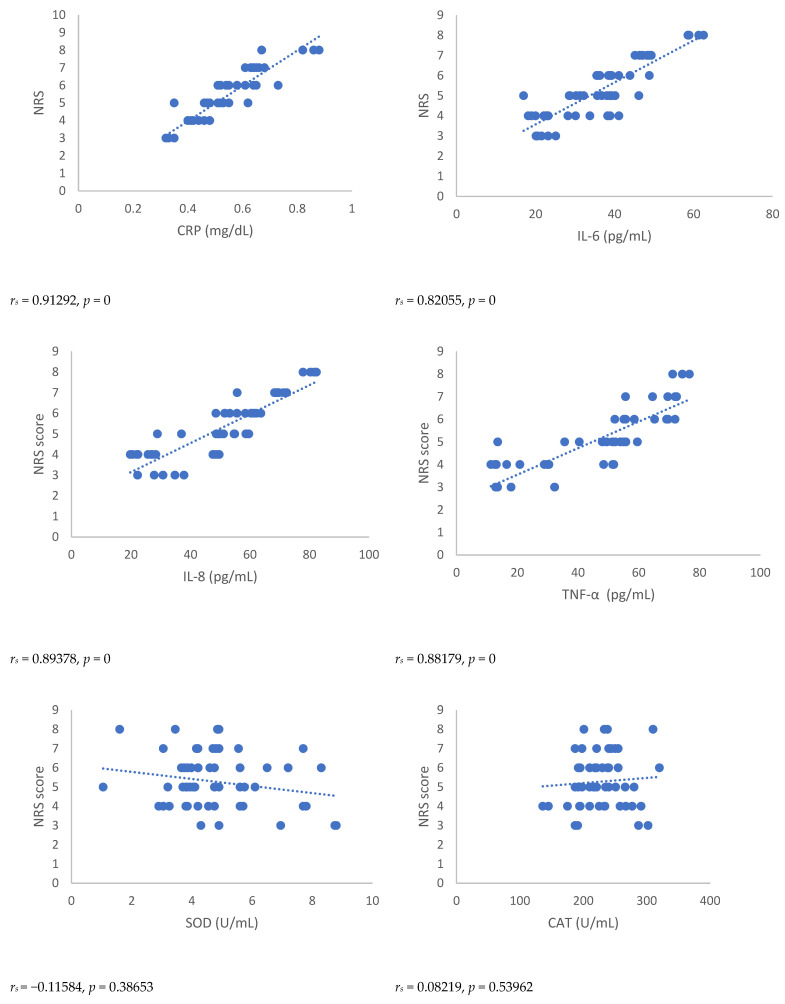

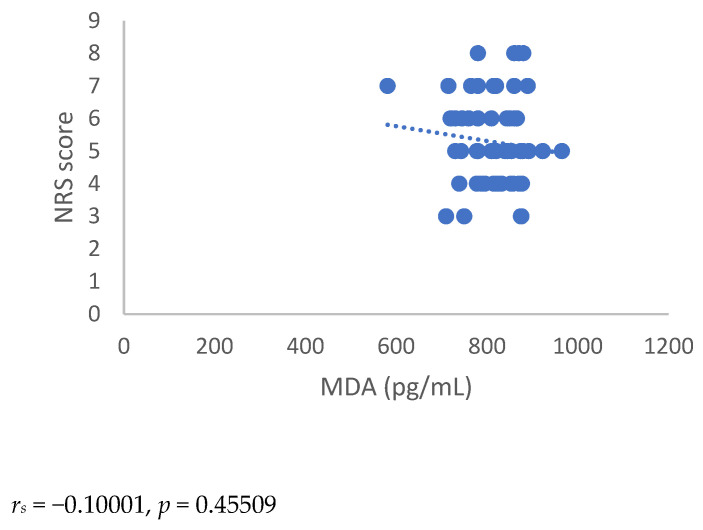
Spearman’s correlation between numerical rating Scale (NRS) score and mediators of inflammation or oxidative stress in the post-mastectomy (PMP) group 3 months after surgery. NRS = Numerical Rating Scale; CRP = C-reactive protein; IL-6 = Interleukin 6; IL-8 = Interleukin 8; TNF-α = Tumor necrosis factor-α; SOD = Superoxide dismutase; CAT = catalase; MDA = Malondialdehyde.

**Figure 2:**
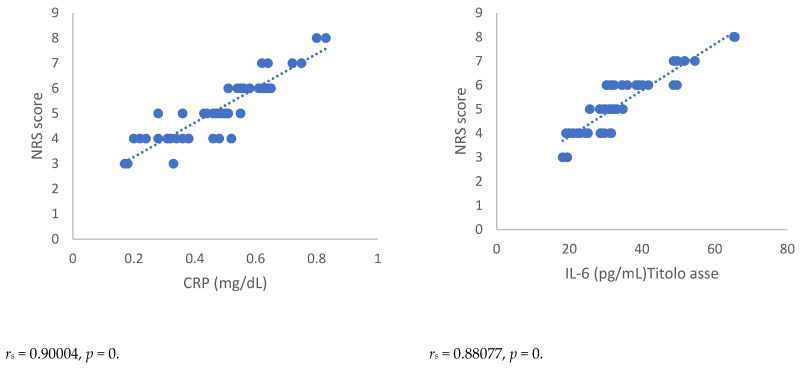

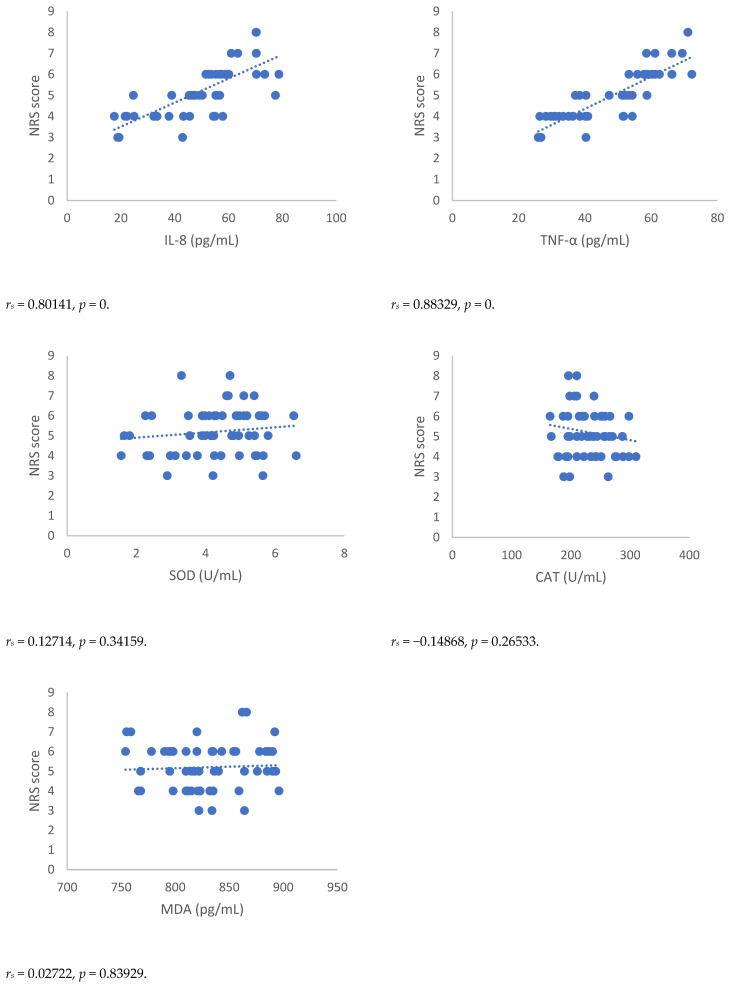
Spearman’s correlation between Numerical Rating Scale (NRS) score and mediators of inflammation or oxidative stress in post-mastectomy (PMP) group, 6 months after surgery. NRS = Numerical Rating Scale; CRP = C-reactive protein; IL-6 = Interleukin 6; IL-8 = Interleukin 8; TNF-α = Tumor necrosis factor-α; SOD = Superoxide dismutase; CAT = catalase; MDA = Malondialdehyde.

**Figure 3:**
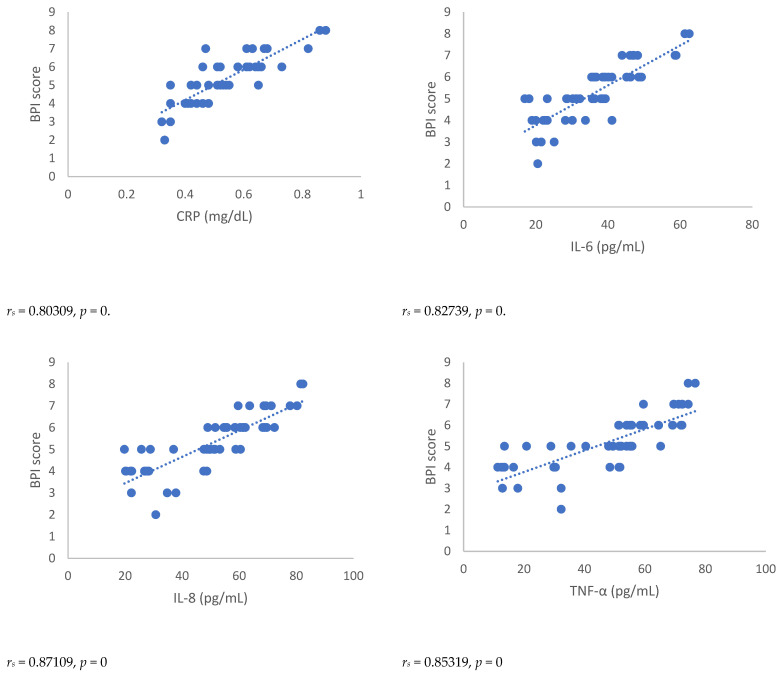

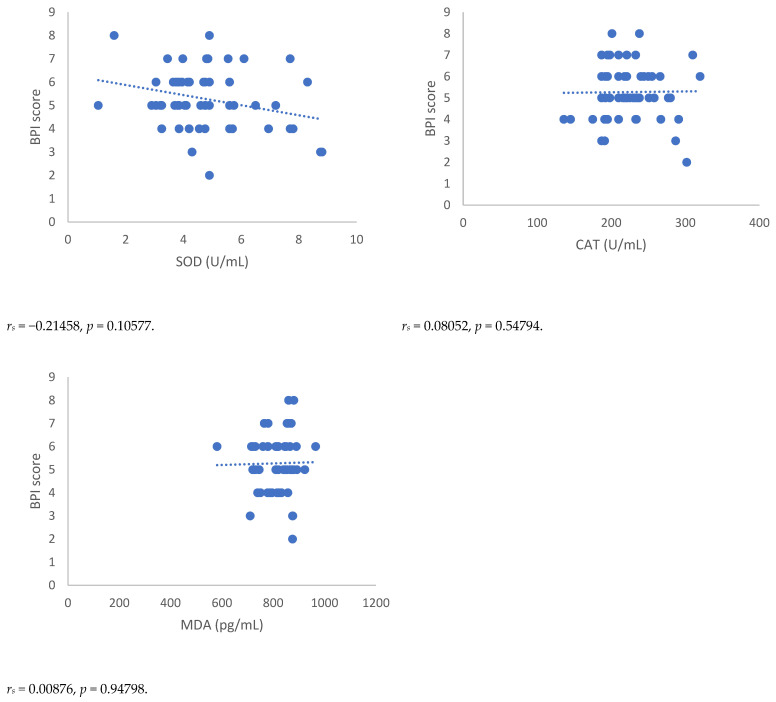
Sperman’s correlation between BPI (Brief Pain Inventory) score and mediators of inflammation or oxidative stress in the post-mastectomy (PMP) group 3 months after surgery. BPI = Brief Pain Inventory; CRP = C-reactive protein; IL-6 = Interleukin 6; IL-8 = Interleukin 8; TNF-α = Tumor necrosis factor-α; SOD = Superoxide dismutase; CAT = catalase; MDA = Malondialdehyde.

**Figure 4:**
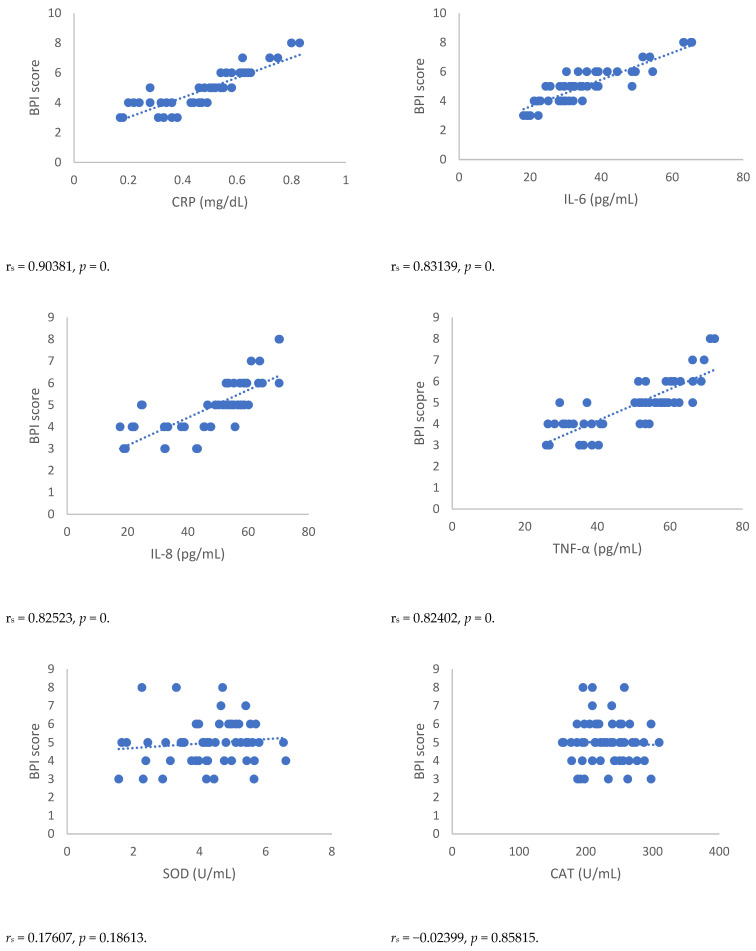

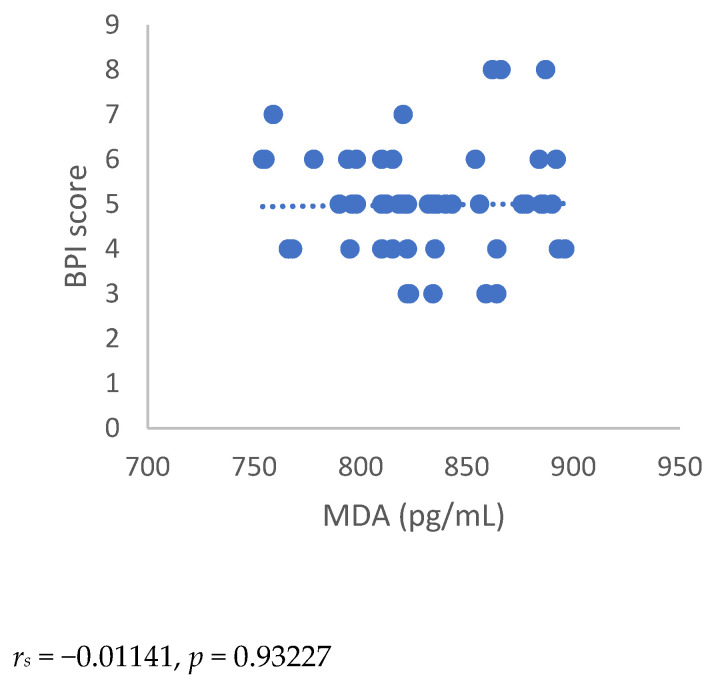
Sperman’s correlation between BPI (Brief Pain Inventory) score and mediators of inflammation or oxidative stress in the post-mastectomy (PMP) group 6 months after surgery. BPI = Brief Pain Inventory; CRP = C-reactive protein; IL-6 = Interleukin 6; IL-8 = Interleukin 8; TNF-α = Tumor necrosis factor-α; SOD = Superoxide dismutase; CAT = catalase; MDA = Malondialdehyde.

**Figure 5:**
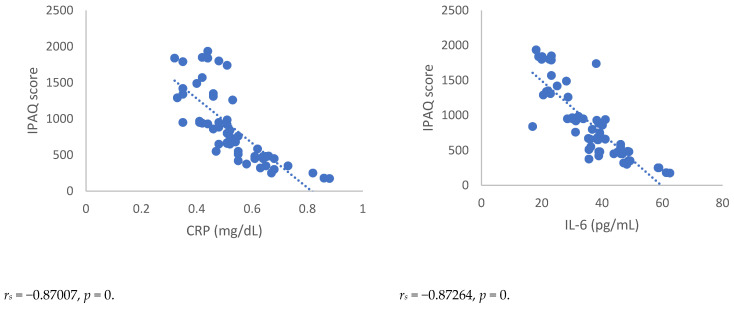

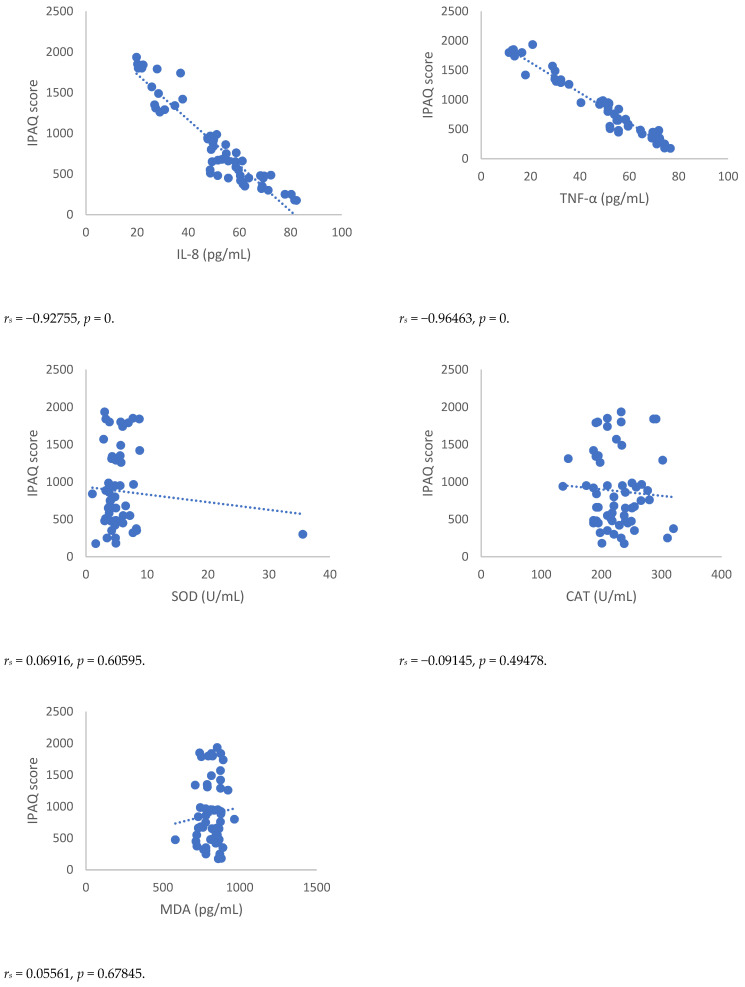
Sperman’s correlation between the IPAQ (International Physical Activity Questionnaire) score and mediators of inflammation or oxidative stress in the post-mastectomy (PMP) group 3 months after surgery. IPAQ = International Physical Activity Questionnaire; CRP = C-reactive protein; IL-6 = Interleukin 6; IL-8 = Interleukin 8; TNF-α = Tumor necrosis factor-α; SOD = Superoxide dismutase; CAT = catalase; MDA = Malondialdehyde.

**Figure 6:**
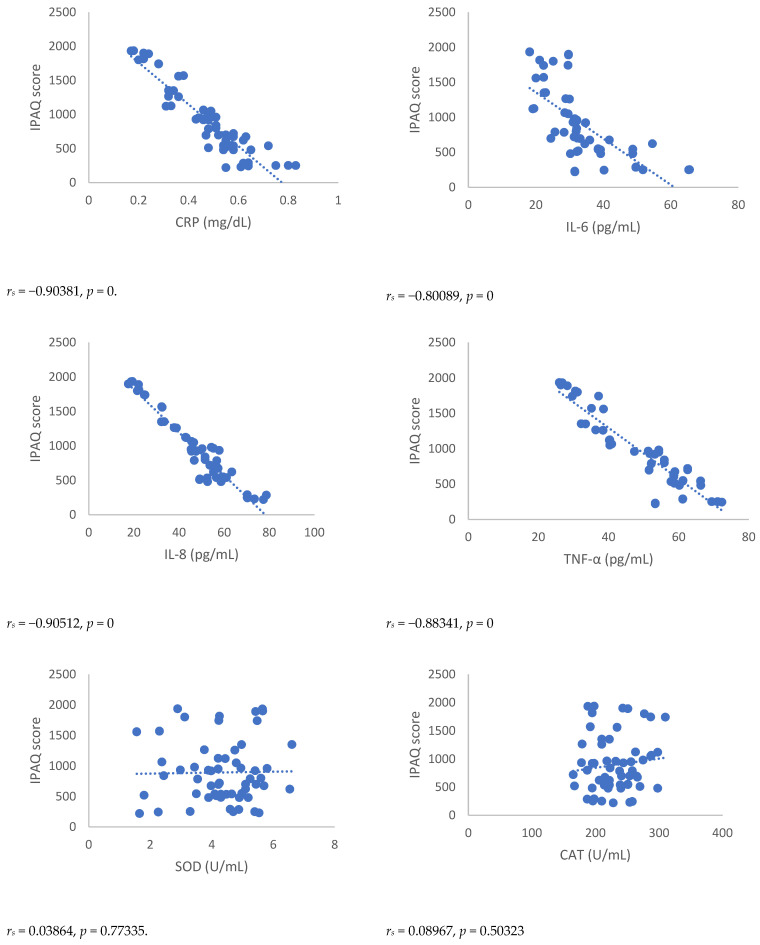

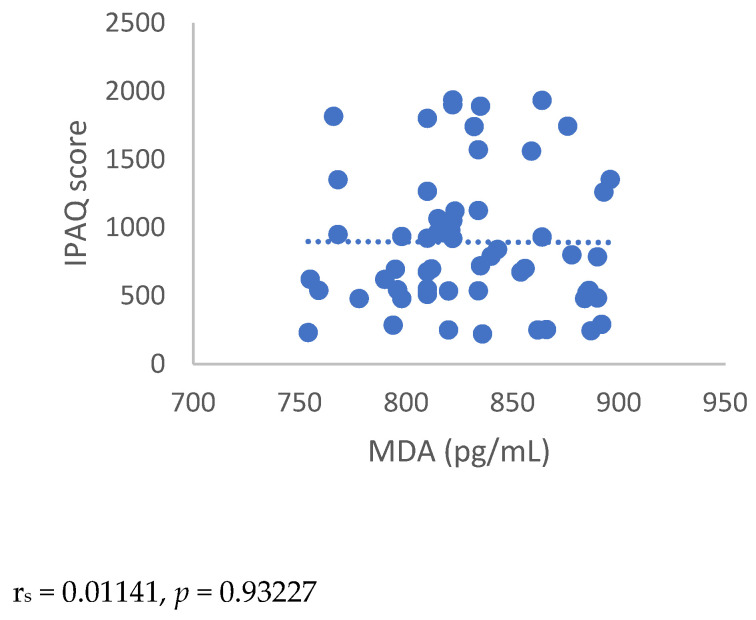
Sperman’s correlation between IPAQ (International Physical Activity Questionnaire) score and mediators of inflammation or oxidative stress in post-mastectomy (PMP) group 6 months after surgery. IPAQ = International Physical Activity Questionnaire; CRP = C-reactive protein; IL-6 = Interleukin 6; IL-8 = Interleukin 8; TNF-α = Tumor necrosis factor-α; SOD = Superoxide dismutase; CAT = catalase; MDA = Malondialdehyde.

**Figure 7:**
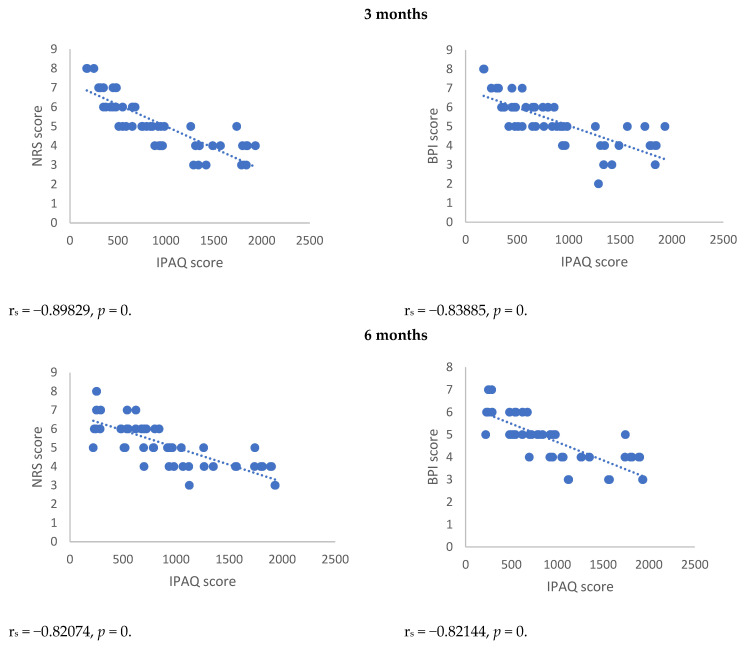
Spearman’s correlation between IPAQ/NRS and IPAQ/BPI in the post-mastectomy (PMP) group 3 and 6 months after surgery. IPAQ = International Physical Activity Questionnaire; NRS = Numerical Rating Scale; BPI = Brief Inventory Pain.

Spearman’s correlation, performed between NRSs or BPI score and mediators of inflammation, showed a statistically significant correlation between NRSs or BPI score and mediators of inflammation both 3 and 6 months after surgery (Figure 1, Figure 2, Figure 3 and Figure 4). Moreover, results showed a negative Spearman’s correlation between IPAQ score and mediators of inflammation (Figure 5 and Figure 6) and between IPAQ and NRS and BPI scores (Figure 7) either at 3 or 6 months after surgery. No correlation between biomarkers of oxidative stress and NRS or BPI or IPAQ scores was detected either 3 or 6 months after surgery (Figure 1, Figure 2, Figure 3 and Figure 4).

## 4. Discussion

Physical activity (PA) is defined as any movement of the body generated by skeletal muscles that requires energy expenditure [26]. Evidence for the efficacy of approaches based on exercise and motor activity in the improvement of pain and function in clinical conditions characterized by chronic pain, such as chronic low back pain, fibromyalgia, osteoarthritis, rheumatoid arthritis, and migraines, has been shown [27]. Moreover, exercise training is also considered safe and beneficial for oncologic patients, who should keep themself physically active [28]. PMP syndrome is characterized by the presence of chronic pain [29,30], and it is estimated that 25–60% of patients who have undergone breast cancer removal surgery suffer from chronic pain [31]. In the present study, 46% of a sample of women who underwent surgery needed for breast cancer removal were shown to be suffering from PMP at 3 and 6 months after the intervention. This proportion is almost overlapping that observed by other authors who reported a pain prevalence of 43% three years after breast surgery [32].

Physical activity evaluation performed by submission of the IPAQ questionnaire to the 58 women affected by PMP recruited in the present study showed that 44.8% of this group was categorized as physically inactive, while 65.5% of the same group was adequately active. According to the answer purchased for IPAQ, no fully active woman was detected in the group and results were overlapping with submission of the questionnaire either at three or six months after surgery. Pain evaluation through NRS and BPI in PMP syndrome women performed at both the scheduled time points (3 and 6 months after surgery) showed a statistically significant increase of both intensity and interference of pain scores in inactive women compared to adequately active women.

Concerning the link between inflammation and pain in people affected by breast cancer, it has been shown that in women affected by this pathology, higher levels of the inflammatory markers C-reactive protein and significant interference of pain with quality of life can be found if compared with the same category of women without pain [33,34]. Other authors suggested the utility of monitoring of pro-inflammatory cytokines by using them as predictors for the severity of pain in breast cancer [35]. In this study, a significant increase in the biomarker of inflammation CRP and of the pro-inflammatory cytokines IL-6, IL-8, and TNF-α can be observed in PMP women compared to non-PMP women either three or six months after surgery.

Evidence shows that exercise has beneficial effects for oncologic patients, and it has been suggested that prevention of physical activity decline and small increases in physical activity levels during and following cancer treatment is appropriate [36]. Furthermore, it has also been speculated that adapted exercise interventions could reduce the possible recurrence of tumor growth in the breast [37]. On this basis, it has been suggested that stretching, gentle aerobic conditioning, low intensity, resistive exercise, and postural modification should be integrated as early as possible after surgery, irrespective of the cancer treatment plan [38].

This beneficial effect can be explained by the knowledge that adequate physical activity increases endogenous antioxidant defenses, counteracting oxidative stress and contributing to the mechanisms of fighting against cancer genesis [39]. In this context, adequate physical activity can improve the health status of patients by enhancing the antioxidative status of the body. In particular, moderate physical activity (chronic exercise or aerobic training) induces a decrease in reactive oxygen species generation improving the antioxidant status [40]. In the sample of women recruited for the present study, a significant reduction of SOD and CAT activity was detected in PMP group of women together with a significant enhancement of MDA at three and six months after surgery, thus indicating an impairment of oxidative balance in women who developed PMP syndrome and suggesting that, in these women, changes in oxidative state could be present.

When pain evaluation data obtained with NRS and BPI are compared with mediators of inflammation through the Spearman’s correlation test, a statistically significant positive correlation between the scores indicating intensity and interference of pain and blood level of markers of inflammation can be noted. Thus, in women in which the scores of NRS and BPI were higher, inflammatory markers such as CRP and pro-inflammatory cytokines were elevated. This correlation was detected either at three or six months after surgery. A negative correlation was observed between IPAQ score towards NRS and BPI scores and between IPAQ score, the blood level of CRP, and mediators of inflammation. This means that the increase of physical activity is associated with a reduction of pain intensity, pain interference, and inflammation, but not with oxidative stress in women affected by PMP syndrome recruited for this study. No correlation between biomarkers of oxidative stress and NRS, BPI, or IPAQ scores was observed either three or six months after surgery.

## 5. Conclusions

These data are interesting, as they show, for the first time, that adequate physical activity can be associated with improvement and reduction of the development of PMP. Moreover, since the level of pain intensity can be used in pain management as a subjective indicator to be quantitatively assessed together with what is generally defined as “interference of pain in activities of daily living” [41], the monitoring of these aspects could be considered a key biomarker for establishing appropriate pain therapy. On this basis, exercise regimens, including the education and promotion of a behavior-reversing against inertia and a sedentary lifestyle favoring the cycle of pain and worsening disability, could be applied in the post-mastectomy period.

## Figures and Tables

**Table 1:** Age, education level, and lymph nodes dissection of the sample of women (n = 126) who underwent a mastectomy.

Group	Age (Years)	Education Level	Lymph Nodes Dissection	Marital Status	NRS
Non-PMP	56.5 ± 10	Secondary = 11.9% Post-secondary = 31.0% Graduation = 11.1%	30.2%	42.9%	1.33 ± 1.73
PMP	56.5 ± 9.7	Secondary = 9.5% Post-secondary = 23.0% Graduation = 13.5%	34.9%	40.5%	5.27 ± 1.38 *

Non-PMP = Non-post-mastectomy pain; PMP = post-mastectomy pain; NRS = Numerical Rating Scale; Mann–Whitney *U* test was used. * = *p* < 0.01 vs. non-PMP; age and NRS data are presented as mean ± standard deviation.

**Table 2:** Evaluation of intensity of pain, interference of pain in daily activities, and biomarkers of oxidative stress and inflammation in post-mastectomy pain (PMP) and non-post-mastectomy pain (non-PMP) groups at 3 and 6 months after surgery.

	Non-PMP n = 68		PMP n = 58	
	3 Months	6 Months	3 Months	6 Months
NRS score (intensity of pain)	1.33 ± 1.73	1.33 ± 1.73	5.27 ± 1.38 *	5.20 ± 1.16 *
BPI score (interference of pain)	0.88 ± 1.26	0.77 ± 1.17	5.27 ± 1.25 *	4.98 ± 1.20 *
CRP (mg/dL)	0.14 ± 0.12	0.16 ± 0.14	0.53 ± 0.12 *	0.49 ± 0.15 *
IL-6 (pg/mL)	13.26 ± 2.11	12.32 ± 2.06	36.30 ± 11.40 *	34.91 ± 11.51 *
IL-8 (pg/mL)	12.44 ± 2.32	12.77 ± 2.31	50.20 ± 17.13 *	48.86 ± 14.26 *
TNF- α (pg/mL)	5.80 ± 2.22	5.6 ± 2.20	49.35 ± 19.37 *	50.84 ± 12.95 *
SOD activity (U/mL)	19.04 ± 1.47	18.54 ± 1.52	5.43 ± 4.35 *	5.81 ± 4.38 *
CAT activity (U/mL)	369.17 ± 1.47	344 ± 1.69	225.70 ± 38.11 *	231.54 ± 37.95 *
MDA (pg/mL)	31.32 ± 2.4	32.66 ± 2.11	801.45 ± 96.93 *	798 ± 96.93 *

NRS = Numerical Rating Scale; BPI = Brief Pain Inventory; CRP = C-reactive protein; IL-6 = Interleukin-6; IL-8 = Interleukin-8; TNF-α = Tumor necrosis factor alpha; SOD = Superoxide dismutase; CAT = catalase; MDA = Malondialdehyde. Data are expressed as mean ± standard deviation. Mann–Whitney *U* test and Wilcoxon tests were used to compare independent groups and paired data, respectively. * = *p* < 0.01 vs. Non-PMP.

**Table 3:** Evaluation of physical activity by IPAQ (International Physical Activity Questionnaire) in the post-mastectomy pain (PMP) group at 3 and 6 months after surgery.

IPAQ Score		
METs	PMP n = 58	
	3 Months after Surgery	6 Months after Surgery
<700 (Inactive) (N = 26)	451.9 ± 152.57	476.3 ± 172.83
700–2509 (Adequate active) (N = 32)	1226.4 ± 445.06 *	1233.9 ± 421.32 *
>2510 (Active) (N = 0)	N.D	N.D.

METs = Metabolic Equivalents of Task; N.D. = not detected. Data are expressed as mean ± standard deviation. Mann–Whitney *U* test was used. * = *p* < 0.01 vs. inactive.

**Table 4:** Evaluation of intensity of pain, interference of pain in daily activities and biomarkers of oxidative stress and inflammation in post-mastectomy pain (PMP) group at 3 and 6 months after surgery, according to physical activity.

	Inactive n = 26		Adequate Active n = 32	
	3 Months	6 Months	3 Months	6 Months
NRS score (Intensity of pain)	6.53 ± 0.85	6.11 ± 0.81	4.25 ± 0.71 *	4.46 ± 0.84 *
BPI score (Interference of pain)	6.15 ± 0.88	5.88 ± 0.99	4.56 ± 1.02 *	4.25 ± 0.80 *
CRP (mg/dL)	0.62 ± 0.10	0.60 ± 0.09	0.45 ± 0.07 *	0.40 ± 0.12 *
IL-6 (pg/mL)	44.91 ± 8.24	43.48 ± 11.23	29.31 ± 8.47 *	27.95 ± 5.30 *
IL-8 (pg/mL)	63.54 ± 10.34	59.33 ± 8.76	39.36 ± 13.52 *	38.56 ± 14.08 *
TNF- α (pg/mL)	64.73 ± 8.12	61.43 ± 5.93	36.85 ± 16.6 *	41.55 ± 11.68 *
SOD activity (U/mL)	4.71 ± 1.46	4.33 ± 1.15	4.84 ± 1.73	4.58 ± 1.67 *
CAT activity (U/mL)	228.34 ± 33.97	223.40 ± 41.02	223.40 ± 41.02	228 ± 29.81 *
MDA (pg/mL)	801.03 ± 70.90	830.76 ± 45.61	827.68 ± 59.33	832.62 ± 38.06 *

Non-PMP = non-post-mastectomy pain; PMP = post-mastectomy pain; NRS = Numerical Rating Scale; BPI = Brief Pain Inventory; CRP = C-reactive protein; IL-6 = Interleukin 6; IL-8 = Interleukin 8; TNF-α = Tumor necrosis factor alpha; SOD = Superoxide dismutase; CAT = catalase; MDA = Malondialdehyde. The Mann–Whitney *U* test and Wilcoxon test were used to compare independent groups and paired data, respectively. Data are expressed as mean ± standard deviation. Inactive < 700 METs; Adequate active = 700–2519 METs; Data are expressed as mean ± standard deviation. * = *p* < 0.01 vs. inactive.

## Data Availability

The data presented in this study are available on request from the corresponding author. The data are not publicly available due to privacy or ethical.
